# Bionomics and vectorial role of anophelines in wetlands along the volcanic chain of Cameroon

**DOI:** 10.1186/s13071-018-3041-z

**Published:** 2018-08-14

**Authors:** Nathalie Amvongo-Adjia, Emmanuela L. Wirsiy, Jacob M. Riveron, Winston P. Chounna Ndongmo, Peter A. Enyong, Flobert Njiokou, Charles S. Wondji, Samuel Wanji

**Affiliations:** 1 Parasitology and Ecology Laboratory, Animal Biology and Physiology Department, Faculty of Science, University of Yaoundé 1, Yaoundé, Cameroon; 2 Research Foundation for Tropical Diseases and the Environment (REFOTDE), Buea, Cameroon; 3 Centre for Medical Research, Institute of Medical Research and Medicinal Plants Studies (IMPM), Yaoundé, Cameroon; 4 Parasite and Vector Biology Research Unit (PAVBRU), Microbiology and Parasitology Department, University of Buea, Buea, Cameroon; 5 Vector Biology Department, Liverpool School of Tropical Medicine, Liverpool, L3 5QA, UK; 6 Centre for Research in Infectious Diseases (CRID), LSTM Research Unit, Yaoundé, Cameroon

**Keywords:** Malaria, *Anopheles* vectors, Volcanic chain of Cameroon, Wetlands, Volcanic massif

## Abstract

**Background:**

The epidemiological profiles of vector-borne diseases, such as malaria, are strongly associated with landscape components. The reduction of malaria burden in endemic and epidemic regions mainly depends on knowledge of the malaria-transmitting mosquito species, populations and behavioural characteristics, as well as malaria exposure risks. This work aimed at carrying out a holistic study in order to characterise *Anopheles* species in relation to human malaria in seven wetlands along the lower section of the volcanic chain of Cameroon.

**Results:**

Eight malaria vectors: *Anopheles arabiensis*, *An. coluzzii*, *An. funestus* (*s.s.*), *An. gambiae*, *An. hancocki*, *An. melas*, *An. nili* and *An. ziemanni*, were found biting humans. *Anopheles gambiae* was widespread; however, it played a secondary role in the Ndop plain where *An. ziemmani* was the primary vector species (79.2%). Anophelines were more exophagic (73.6%) than endophagic (26.4%), showing a marked nocturnal activity (22:00–4:00 h) for *An. coluzzii* and *An. gambiae* while *An. funestus* (*s.s.*) was mostly caught between 1:00 and 6:00 h and *An. ziemanni* having an early evening biting behaviour (18:00-00:00 h). Female *Anopheles* were mostly observed to have relative high parity rates (≥ 70%), with the exception of the Meanja site where species parity varies from 46 to 55%. Overall, the transmission level was low with entomological inoculation rates estimated to 0.7 infected bites per person per month (ib/p/mth) in Tiko and Ndop, 1.4 ib/p/mth in Mamfe and 2.24 ib/p/mth in Santchou.

**Conclusions:**

The present study represents detailed *Anopheles* vector characterisation from an understudied area along the volcanic chain of Cameroon with endemic malaria transmission. The significant differences in bionomics and *Anopheles* species distribution within the studied wetlands, highlights the importance of providing baseline data and an opportunity to assess the outcome of ongoing malaria control interventions in the country.

**Electronic supplementary material:**

The online version of this article (10.1186/s13071-018-3041-z) contains supplementary material, which is available to authorized users.

## Background

Malaria, transmitted by the bite of infected female *Anopheles* mosquitoes, is a major health burden in the Cameroon hill tracts [1–5]. The area has an estimated *Plasmodium falciparum* prevalence rate (*Pf*PR) ranging from 50 to 70%, thus the disease remains the leading cause of consultation in health services [6, 7]. Although there are 48 *Anopheles* species recorded throughout Cameroon, only 18 are responsible for malaria transmission [8–11]. These include: *Anopheles arabiensis*, *An. coluzzii*, *An. gambiae* and *An. funestus* (*s.s.*) as dominant vectors species, while *An. carnevalei*, *An. coustani*, *An. hancocki*, *An. leesoni*, *An. marshallii*, *An. melas*, *An. moucheti*, *An. nili*, *An. paludis*, *An. pharoensis*, *An. ovengensis*, *An. rivulorum-like*, *An. wellcomei* and *An. ziemanni* play a secondary role.

Various factors contribute to differing malaria epidemiological profiles including altitude, topography, hydrology and land use/land cover types [12, 13]. Specifically, changes of environmental factors can impact malaria transmission by altering the microclimate of the immature stages and adult mosquitoes [14, 15], as it has been observed that the shortening of *Plasmodium* sporogony and vector gonotrophic cycle lead to an increase of the malaria transmission risk across highlands [16]. Furthermore, malaria prevalence is related to the existence of wetlands or swampy areas, because mosquitoes require stagnant water as their larval habitat [17, 18]. In fact, along the volcanic chain of Cameroon (VCC), there exist a considerable number of wetlands in valley bottoms of mountain massifs that are maintained by high annual precipitation and favourable surface hydrology [19]. Of further note, these volcanic mountains possess highly fertile and productive soils resulting in a concentration of human populations [20, 21].

The level of malaria transmission is determined by the interactions between *Plasmodium* parasites, the *Anopheles* vectors and the human host. Understanding adult vector population dynamics by identifying the different species, their abundance, biting behaviour and entomological inoculation rates are important steps towards effective control of malaria, with vector abundance being a key determinant of malaria transmission force [22]. Potential reductions of the malaria burden in endemic and epidemic regions depends on knowledge of the malaria-transmitting mosquito species, populations and behavioural characteristics, and malaria exposure risks. While vector populations have been extensively studied in urban [23–25], forested [26–28] and savannah [9, 29–31] areas of Cameroon, only a few studies on malaria vector populations have been conducted in some isolated wetlands across the VCC [1, 2, 4, 5]. This disparity in the level of knowledge of the malaria vectorial system (species composition, feeding behaviour, biting cycle, entomological indices of transmission) between the Cameroon hill tract and the rest of the country is mainly due to the difficult access towards those areas. In this study, sampling was carried out in seven wetlands along the lower section of the VCC in order to assess the bionomics and vectorial role of *Anopheles* species in relation to human malaria.

## Methods

### Study sites

The volcanic chain of Cameroon is a 1600 km long massif of cenozoic volcanic and subvolcanic complexes that straddles the continent-ocean boundary. It extends from the Gulf of Guinea to the interior of the African continent. In Cameroon, it starts with Mount Cameroon, extends to the Western High Plateau and northeast towards Lake Chad (see Additional file 1: Figure S1). The line strikes almost south-west to north-east, forming a swell and basin structure where the six islands and the four central volcanoes of Mount Cameroon, Kupe Manengouba, Bamboutous, Bamenda and Oku represent swells. The presence of these large land masses and several volcanic cones facilitate the occurrence of orographic uplift, which accounts for the intensive precipitation resulting on a major input water into the wetlands [32].

**Fig. 1:**
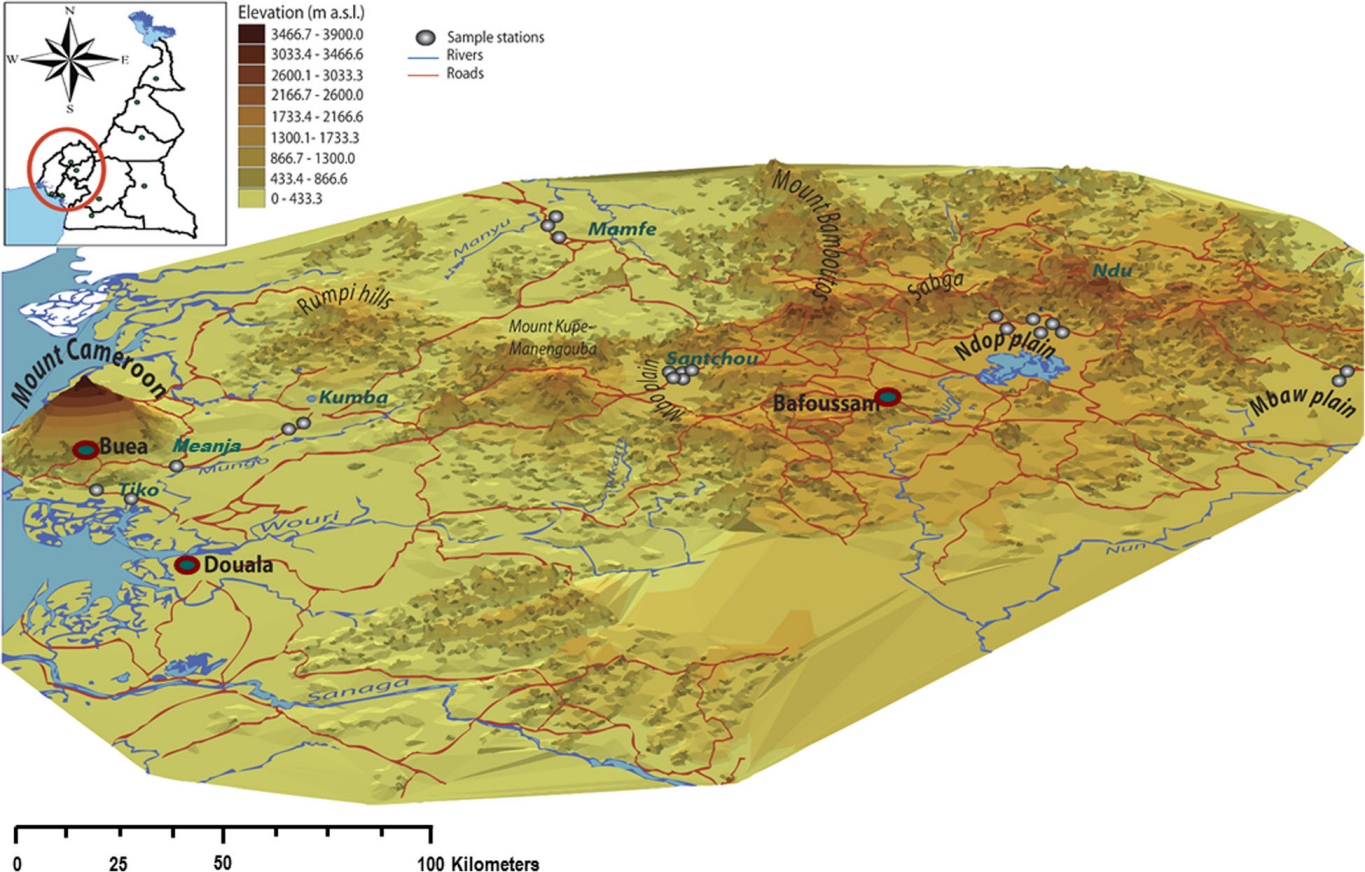
3D map of the study area

Many wetlands exist in Cameroon, with some located inland and across the national frontier, and others located within the coastal lowlands of mountain massifs. Some of these wetlands result from lava filled depressions, topographic channels which widen into flood plains and braided profiles, tidal activities in estuarine environments and shallow depressions beside the coast [33, 34]. For the current study, seven wetlands surrounded by five volcanic massifs along the lower section of the Cameroon volcanic line were selected (Fig. 1, Additional file 2: Table S1). These were: (i) Mount Cameroon massif: Tiko [4°07'N, 9°36'E, 64 m above sea level (masl)], Kumba (4°38'N, 9°27'E, 240 masl) and Meanja (4°43'N, 9°38'E, 305 masl); (ii) Western Highlands: Mamfe (5°46'N, 9°17'E, 126 masl); (iii) Mount Kupe Manengouba: Santchou (5°15'N, 9°50'E, 750 masl); (iv) Bamboutos: Ndop plain (5°56'N, 10°32'E, 1152 masl); and (v) Mount Oku/Ndu massif: Mbaw plain (5°55'N, 10°10'E, 400 masl).

### Study design and adult mosquito sampling

Repeated cross-sectional surveys were carried out to conduct this study (see Additional file 3: Figure S2). Mosquito collections were undertaken using landing catches on human volunteers (human-landing catch: HLC) during the rainy season months of April and May 2010 (beginning of rainy season for Tiko, Meanja and Santchou), September and October 2013, and October 2014 (end of rainy season for Kumba, Mamfe, Ndop and Mbaw plain). Depending on size of the volcanic massifs and/or wetlands, one to six communities were chosen as sampled stations. During five consecutive nights (from 18:00 to 6:00 h), HLCs were performed in two houses per community with one collector indoors and one outdoors (approximately 5 m from the main entrance) selected households (see Additional file 4: Table S2). Householders were asked to assist designated mosquito collectors (four men per night per sampled station) in each community for 5 days per study site. Using this strategy, the *Anopheles* species man biting rate (MBR) or *Anopheles* density per person per time unit (month) was estimated and used for the calculation of the entomological inoculation rates (EIR). All mosquitoes collected were placed in holding cups labelled by hour until they were processed for morphological identification.

### Morphological identification of *Anopheles* species and age-grading methods

After being differentiated at the level of genus as *Culex*, *Anopheles*, *Aedes* or *Mansonia*, *Anopheles* mosquitoes were identified to species level using the taxonomic and identification key to female Afrotropical anophelines [35, 36]. Age-grading was based on the mosquitoes’ physiological age. More precisely, the physiological age was determined from inspecting its ovaries, based on the ovary tracheation method of Detinova [37] and as described by the simplified technique of Warrel & Gilles [38]. This dissection technique of the ovaries can differentiate parous female from nulliparous. Female *Anopheles* that have taken a blood meal and have laid their eggs at least once are parous, while those that have never laid eggs are nulliparous.

### DNA isolation, species identification and molecular detection of *Plasmodium* spp. infection in *Anopheles* mosquitoes

Genomic DNA was individually isolated from most recurrent *Anopheles* specimens using a DNA extraction buffer [38]. Species were molecularly identified using sequences of the ribosomal DNA intergenic spacer (rDNA IGS) and internal transcribed spacer region 2 (ITS2) of *An. gambiae* (*s.l*.) and *An. funestus* (*s.l.*), respectively. These regions were isolated and amplified using PCR with IGS and ITS2 specific primers as previously documented [39, 40].

A restricted fragment length polymorphism (RFLP) was further performed on all the specimens diagnosed as *An. gambiae* (*s.s.*) to distinguish between molecular forms M and S [41]. The M molecular form of *An. gambiae* (*s.s.*) is presently known as *An. coluzzii* while the S molecular form retained the designation *An. gambiae* [42].

The infection status of female *Anopheles* was determined using a multiplex PCR for *P. falciparum*, *P. malariae*, *P. vivax* and *P. ovale*. Oligonucleotides were designed by Snounou et al. [43] and Padley et al. [44].

### Data processing and statistical analysis

Data were compiled in EpiInfo^TM^ v.3.5.3 (Centre for Disease Control and Prevention, Atlanta, USA) and imported to SPSS v.20 (IBM, Chicago, USA) and GraphPad Prism v.7.0.0 for Windows (La Jolla, California, USA) for analysis. The data of malaria vector density (including diversity, biting density, biting behaviour and biting cycle) were summarized using descriptive statistics. Chi-square test was used to compare mosquito density distributions between volcanic massifs and wetlands. T-test and two-way ANOVA (on normalized data using the logarithm procedure) followed by Bonferroni’s multiple comparison test were used to compare the mean indoor and outdoor exposure to main *Anopheles* vectors within and between wetlands.

Mosquito life expectancy (longevity, life span) was estimated using Davidson formula [45]:

$$ Age=\frac{1}{-{\log}_{e^P}} $$

where “e” is the natural logarithm of the constant 2.7183 and *P* is the probability of the vector surviving for 1 day. *P* is calculated from the following formula:

$$ P=\sqrt[ gc]{PR} $$

where gc is the duration of the *Anopheles* gonotrophic cycle, the time (days) needed by a female mosquito to complete the processing of egg development in the ovaries from the blood meal to the time when the fully developed eggs are laid [46]. The parity rate (PR) is the rate of parous female *Anopheles* from the total number of ovaries dissected and observed. In this study, the gc anthropophagous species was 3 days while zoophagic species had a gc of 2 days [47]. The parity rate between *Anopheles* species and surveyed sites was compared using the Chi-square test.

The entomological inoculation rate (EIR) was calculated using the formula:

$$ EIR= IR\times MBR $$

where IR is the rate of *Anopheles* tested positive for the molecular detection of *Plasmodium* spp. infection, and MBR the man biting rate expressed as the ratio of the number of *Anopheles* mosquitoes captured by HLC by the total number of man-nights.

## Results

### Survey summary

**Fig. 2:**
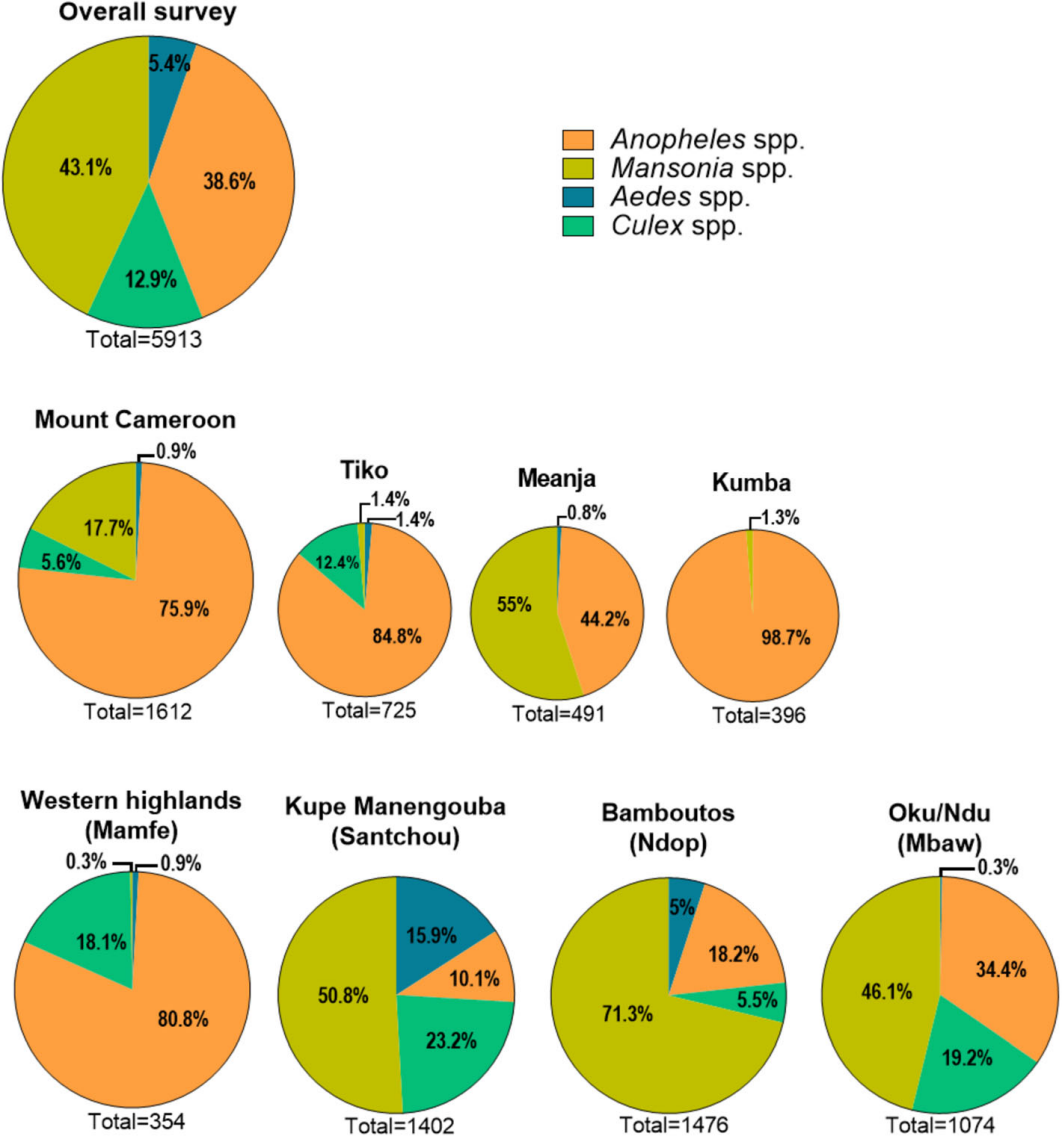
Main genera of mosquitoes identified morphologically from wetlands of the different massifs of the volcanic chain of Cameroon. A significant difference based on a Chi-square test (*P* < 0.0001) was noted in the mosquito distribution for all sites

A total of 5918 mosquitoes were collected during the entire survey period. Four genera were recorded based on the morphological identification of the specimens (Fig. 2): *Aedes* (5.4%), *Culex* (12.9%), *Anopheles* (38.6%) and *Mansonia* (43.1%). The Mount Cameroon massif had the highest number of *Anopheles* specimens caught (53.5%) while the Santchou valley in the Kupe Manengouba massif had the lowest contribution (6.2%). Except in Mount Cameroon (Tiko, Kumba, Meanja) and Western Highlands (Mamfe) that were dominated by *Anopheles* mosquitoes (75.9 and 80.8%, respectively), mosquitoes of the genus *Mansonia* was dominant in the Kupe Manengouba, Bamboutous and Oku volcanic massifs. For these last study sites, catches of *Anopheles* mosquitoes represented 10.1, 18.2 and 34.4%, respectively.

### Anopheline species diversity and biting density

**Fig. 3:**
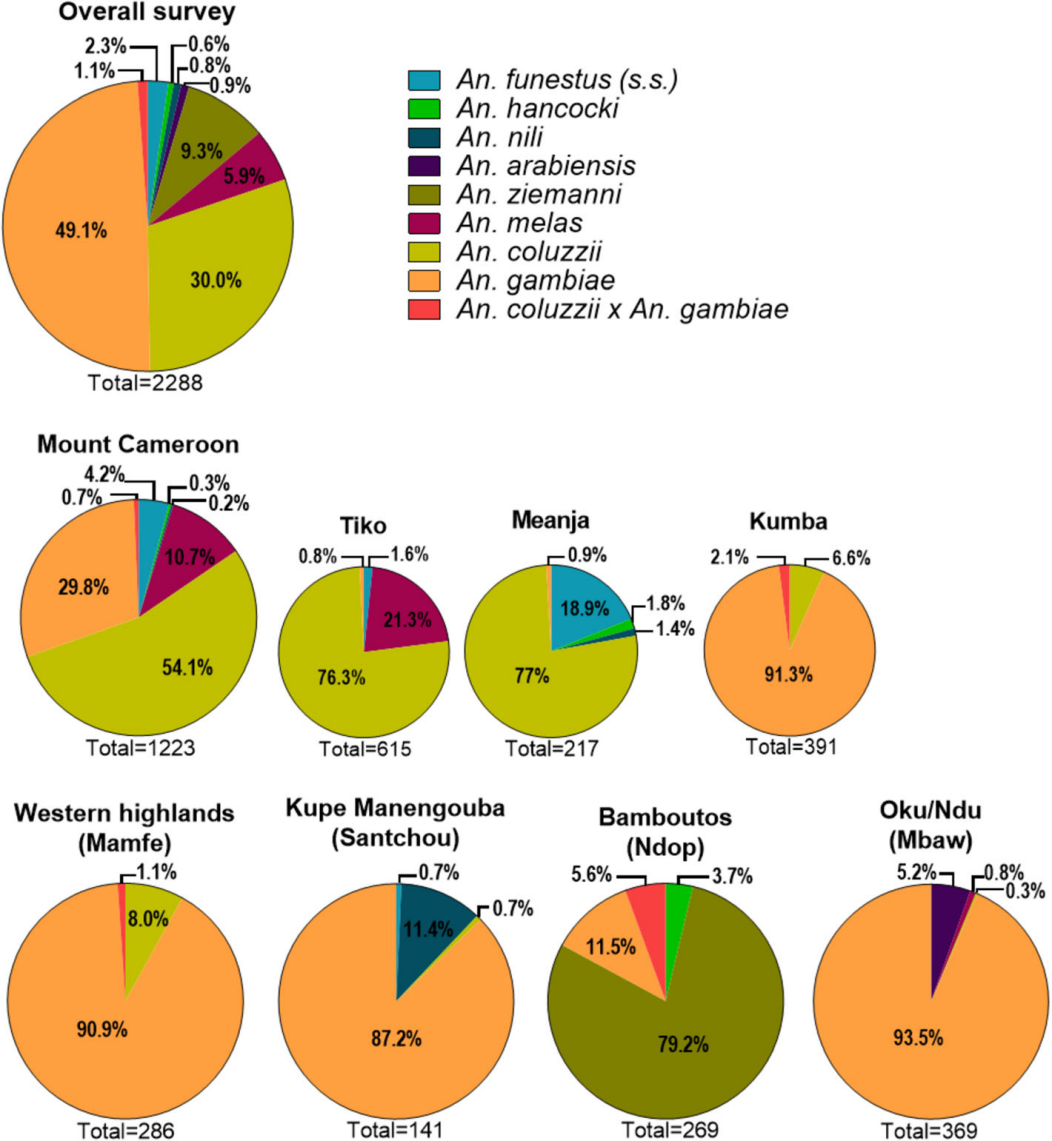
Diversity of *Anopheles* species (identified morphologically and using molecular techniques) from wetlands of major volcanic massifs across the study area. A significant difference based on a Chi-square test (*P* < 0.0001) was noted in anopheline abundance in all sites

Five *Anopheles* morphological species leading to eight molecular species were identified (Fig. 3): *An. hancocki* (n = 14 specimens caught), *An. nili* (n = 19 specimens caught), *An. ziemanni* (n = 213 specimens caught), within the group of sister species *An. funestus* (*n* = 52 specimens caught), the sole species identified molecularly was *An. funestus* (*s.s.*) and within the complex *An. gambiae* (*s.l.*) (*n* = 1990 specimens caught), four species were identified throughout this study: *An. arabiensis* (1.0%), *An. melas* (6.7%), *An. coluzzii* (34.5%) and *An. gambiae* (56.4%). Additionally, some hybrids *An. coluzzii* × *An. gambiae* (1.3%) were also molecularly identified within the Gambiae complex in Kumba, Mamfe and Ndop sites.

*Anopheles coluzzii* was dominant in the western part of the study area, especially towards the sea at Tiko and Meanja, whereas *An. gambiae* was dominant in other sites except for the Ndop site where *An. ziemanni* was widely represented among night HLCs. The Mount Cameroon area had the highest species diversity [six *Anopheles* species out of the eight identified: *An. funestus* (*s.s.*), *An. hancocki*, *An. nili*, *An. melas*, *An. coluzzii* and *An. gambiae*] while only two *Anopheles* species (*An. coluzzii* and *An. gambiae*) were found in Mamfe towards the Western Highlands. Overall, it was noted a significant difference in the diversity of the different anopheline vectors across the study area (*χ*^2^ = 4520.1, *df* = 48, *P* < 0.0001).

### Biting behaviour

**Fig. 4:**
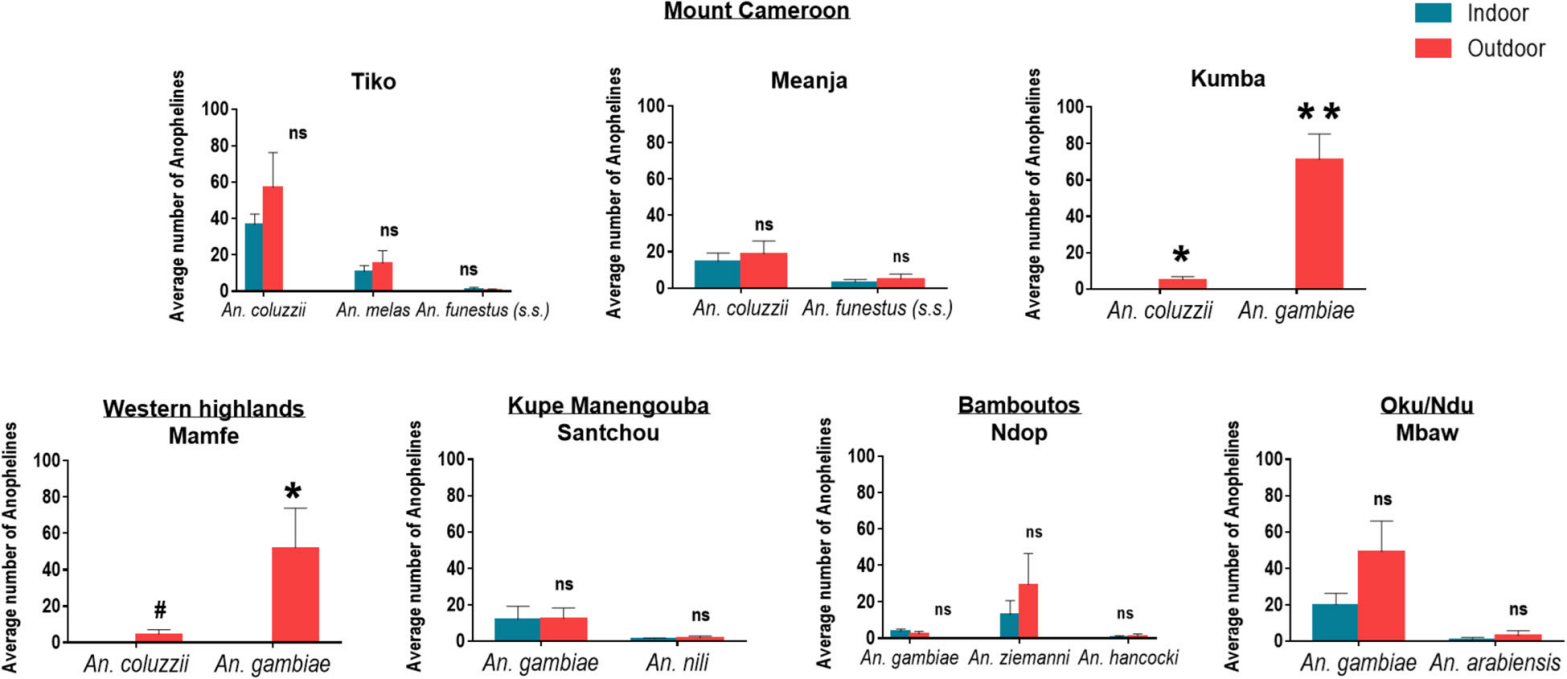
Indoor and outdoor exposure to major anophelines per wetland. Anophelines were mostly collected outdoors; note that there were no specimens collected indoors in Kumba and Mamfe. t-test statistical significance was determined using the Boferonni-Dunn method for each *Anopheles* species biting behaviour per wetland. ^#^*P* = 0.0986, *P < 0.05, **P < 0.001. *Abbreviation*: ns, not significant

The HLC collections indicated indoor and outdoor exposure of residents to host-seeking anophelines. In this study 1683 (73.6%) of the total female *Anopheles* specimens collected were captured outdoors, showing a strongly exophagic biting behaviour. Two-way ANOVA test showed some significant differences in the mean number of *Anopheles* species caught outdoors within wetlands as well as between wetlands (see Additional file 5: Table S3), whereas only 605 (26.4%) anophelines were captured indoors with no significant difference observed in the average number of *Anopheles* species caught within and between wetlands. Moreover, no statistical difference between indoor and outdoor exposure to *Anopheles* species was usually noted in the surveyed wetlands, except in the cities of Kumba and Mamfe where all specimens of the Gambiae complex were collected outside the houses (Fig. 4).

**Fig. 5:**
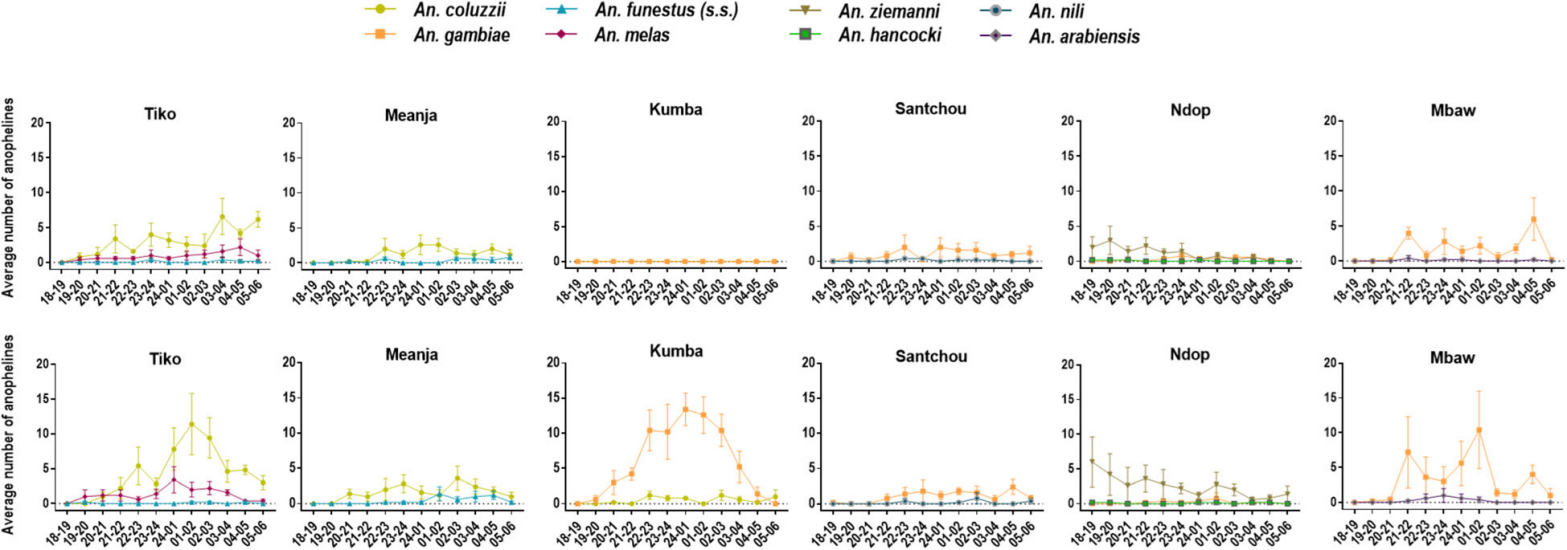
Biting cycles of most predominant anophelines collected indoors (upper panels) and outdoors (lower panels) per wetland. *An. coluzzii* and *An. gambiae* were the most abundant specimens sampled both indoors and outdoors; *An. ziemanni* bites early in the evening

Comparison of anophelines biting cycle showed that dominant vectors (*An. gambiae* and *An. coluzzii*) were collected both indoors and outdoors with a significant number of bites registered late at night between 22:00 h and 4:00 h (*An. coluzzii*: *χ*^2^ = 417.6, *df* = 2, *P* < 0.0001; *An. gambiae*: *χ*^2^ = 588, *df* = 2, P < 0.0001) as compared to *An. funestus* (*s.s*.) which was mostly (*χ*^2^ = 19.69, *df* = 1, *P* < 0.0001) captured between 1:00 h and 6:00 h (Fig. 5). Indoor species were captured between 21:00 h and midnight. Conversely, *An. ziemanni* had an early evening biting behaviour (18:00–00:00 h) both indoors and outdoors (*χ*^2^ = 59.95, *df* = 1, *P* < 0.0001).

### *Anopheles* parity rate and longevity

The parity rate (parous ratio: PR) and life expectancy or life span were analysed for Tiko (*An. melas* and *An. coluzzii*), Meanja (*An. funestus* and *An. coluzzii*), Santchou (*An. gambiae*) and Ndop (*An. ziemanni* and *An. gambiae*). PR was used to estimate mosquito longevity based on the formula proposed by Davidson [45]. Since there was no direct observation of the gonotrophic cycle (gc) value, the mosquito life span calculation used a gc value of 2 or 3 days as previously explained.

Overall, parity ratios varied from 0.55 in *An. funestus* (*s.s.*) to 0.85 in *An. ziemanni*, whereas *An. coluzzii*, *An. gambiae* and *An. melas* had an estimated parity of 0.67, 0.73 and 0.79, respectively (Table 1). Likewise, throughout the study area, species life expectancies estimates were: 5.02 days in *An. funestus* (*s.s.*), 7.49 days in *An. coluzzii*, 9.53 days in *An. gambiae*, 12.31 days in *An. ziemanni* and 12.73 days in *An. melas*. No statistical difference was observed in the distribution of parity rate between species; however, only *An. coluzzii* exhibited significant different parous rates per site (*χ*^2^ = 10.25, *df* = 1, *P* = 0.0014) while comparing the Tiko and Meanja surveys.

**Table 1:** Parous rate and duration of life expectancy of *Anopheles* species from the different study sites

Volcanic massif	Wetland^a^	Species	Dissected	Parous	PR	*P* ^b^	Life span (days)
Mount Cameroon	Tiko	*An. melas*	99	78	0.79	0.92	12.73
		*An. coluzzii*	337	261	0.77	0.92	11.48
	Meanja	*An. funestus* (*s.s.*)	38	21	0.55	0.82	5.02
		*An. coluzzii*	157	72	0.46	0.77	3.86
Kupe Manengouba	Santchou	*An. gambiae*	116	80	0.69	0.88	8.08
Bamboutos	Ndop	*An. ziemanni*	181	154	0.85	0.92	12.31
		*An. gambiae*	31	28	0.90	0.97	28.47

^a^No *Anopheles* dissected in Kumba, Mamfe and Mbaw plain

^b^No statistical difference was observed in the distribution of parity rate between species; however, a significant difference (Chi-square test: *P* < 0.05) was noted in the *An. coluzzii* parous rate while comparing Tiko and Meanja sites

*Abbreviations*: *PR* parous ratio, *P* probability of daily survival^.^

### *Plasmodium* infection and entomological inoculation rates

A total of 2209 *Anopheles* mosquitoes [20 *An. arabiensis*, 51 *An. funestus* (*s.s.*), 131 *An. melas*, 213 *An. ziemanni*, 685 *An. coluzzii* and 1109 *An. gambiae*] were tested for *Plasmodium* infection using a TaqMan assay. Only three *Anopheles* species were found positive during the molecular detection of *Plasmodium* infection, namely *An. coluzzii* (0.15%), *An. gambiae* (1.08%) and *An. ziemanni* (1.41%) (Table 2). Infected *Anopheles* were found only in four wetlands out of the seven surveyed: Tiko (*An. coluzzii*), Mamfe (*An. gambiae*), Santchou (*An. gambiae*) and Ndop (*An. gambiae* and *An. ziemanni*). Infection rates (IRs) varied between *Anopheles* species and wetlands and ranged between 0–6.9% with no statistical difference (based on Chi-square tests) found between anophelines and wetlands. *Anopheles coluzzii* was the species most commonly found biting humans with a man biting rate (MBR) of 328.3 bites per person per month (b/p/mth) while for *An. gambiae* it ranged from 7.2 b/p/mth in Ndop to 121.3 b/p/mth in Mamfe, and *An. ziemanni* had 49.7 b/p/mth. Thus, the level of overall malaria transmission varies between *Anopheles* species and wetlands. *An. gambiae* appears to be the most frequently competent malaria vector with entomological inoculation rates (EIRs) estimated at 0.23 infected bites per person per month (ib/p/mth) in Ndop, 1.4 ib/p/mth in Mamfe and 2.24 ib/p/mth in Santchou. However, similar EIRs (0.7 ib/p/mth) were observed for *An. coluzzii* in Tiko and *An. ziemanni* in Ndop.

**Table 2:** Infection rate and weekly entomological inoculation rate of *Anopheles* vectors in the study site

Volcanic massif	Wetland	Species	Tested	Positive	IR (%)	MBR (b/p/mth)	EIR (ib/p/mth)
Mount Cameroon	Tiko	*An. coluzzii*	469	1	0.21	328.3	0.70
		*An. melas*	131	0	0	91.7	0
		*An. funestus* (*s.s.*)	10	0	0	7.0	0
	Meanja	*An. coluzzii*	167	0	0	116.9	0
		*An. funestus*	41	0	0	28.7	0
	Kumba	*An. coluzzii*	26	0	0	18.2	0
		*An. gambiae*	357	0	0	249.9	0
Western Highlands	Mamfe	*An. coluzzii*	23	0	0	16.1	0
		*An. gambiae*	260	3	1.15	121.3	1.40
Kupe Manengouba	Santchou	*An. gambiae*	116	8	6.90	32.5	2.24
Bamboutos	Ndop	*An. gambiae*	31	1	3.22	7.2	0.23
		*An. ziemanni*	213	3	1.41	49.7	0.70
Oku/Ndu	Mbaw	*An. arabiensis*	20	0	0	4.7	0
		*An. gambiae*	345	0	0	80.5	0

*Abbreviations*: *IR* infection rate, *MBR* man biting rate as estimated by the number of bites per person per month (b/p/mth), *EIR* entomological inoculation rate as the number of infected bites per person per month (ib/p/mth)

## Discussion

Malaria remains a significant public health problem in Cameroon. Using standard procedures of collecting and processing samples, this study demonstrates the wide range of vector composition and distribution, exposure in sampled sites, and entomological indices of transmission in seven wetlands along the downer section of the VCC.

During this study, *Anopheles* and *Mansonia* were the most predominant culicid genera found throughout the study area. The Mount Cameroon massif contributed up to 75.9% of the entire *Anopheles* fauna collected while *Mansonia* species were mostly associated with rice flood plains (Santchou, Ndop and Mbaw). These observations were contrary to other studies which associated *Anopheles* mosquitoes with a wide range of habitats including rice field ecosystems [48–50], whereas *Mansonia* species showed a preference to ponds infested with *Pistia* spp. which are also common in such areas [51]. In addition to the species of these genera, *Aedes* spp. and *Culex* spp. were also encountered in wetlands of the VCC. In Africa, these mosquitoes are known to transmit *Wuchereria bancrofti* microfilariae, the causative agents of lymphatic filariasis in Africa (mainly *Anopheles* spp. and *Culex* spp.), and viruses causing dengue fever and yellow fever (*Aedes* spp.) as well as Rift Valley fever (*Culex* spp. and *Aedes* spp., but also *Anopheles* spp. and *Mansonia* spp. in the enzootic cycle in East Africa).

All five morphological malaria vector taxa (leading to eight molecular species) recorded in Cameroon [8–11] were found biting humans along the volcanic chain of Cameroon. High species diversity was noted in the Mount Cameroon massif (6 out of 8 species), Kupe Manengouba and Oku massifs (4 out of 8 species each), whereas only two *Anopheles* species were found in the Western Highlands and Oku massifs, with observations being in accordance with previous reports [1, 4, 5].

The most widespread and competent malaria vector in sub-Saharan Africa, *An. gambiae*, was found to be present throughout the studied wetlands. Its distribution and predominance are consistent with previous studies [1, 9, 52, 53]. The adaptability of this species to different topographic settings has also been widely reported [4, 10]. In Ndop, although *An. gambiae* and *An. ziemanni* were both involved in malaria transmission, the dominant vector was *An. ziemanni*. Here, the high density of *An. ziemanni* was probably a consequence of the ecosystem consisting of paddy fields irrigated by water (vegetative swamps) sourcing from numerous natural lakes, which enables the proliferation of breeding sites that favour its development [5, 54]. The localised predominance of *An. ziemanni* has been previously observed in the same region [5], as well as in western Kenya [55] and south-central Ethiopia [54] where the swamps contain a lot of aquatic vegetation as with Ndop plain.

The two sibling species of *Anopheles coluzzii* and *An. gambiae* were spatially segregated across the study sites. This is in-line with observations made by other studies [42, 56] which report that these molecular forms are cohesive and constitute exclusive taxonomic groups across their shared range. *An. coluzzii* is more adapted to urbanized and polluted environments (such as Tiko and Meanja) [57], and environments having high salinity rates [58], while *An. gambiae* seems predominant in semi-urban, rural and arid environments (such as Kumba, Mamfe, Santchou and Mbaw plain) [59]. Hybridization between forms occurs rarely (~1%) in Central Africa [60, 61] as confirmed in our study where a low rate of *An. coluzzii* × *An. gambiae* hybrids was noted. Furthermore, 5.6% of *An. coluzzii* × *An. gambiae* hybrids in the Ndop area was noted, whereas only *An. gambiae* molecularly identified, opposing observations made by Tabue et al. [5] who reported *An. coluzzii* in the same region. Nevertheless, this difference could be justified by the fact that during this study investigators surveyed different sampled stations than the previous team. Thus, more studies should be conducted in the Ndop area in order to understand the distribution of *An. gambiae* (*s.s.*) (formerly grouping *An. gambiae* M and S molecular forms) species.

Other malaria vectors such as *An. funestus* (*s.s.*), *An. nili* and *An. hancocki* were recorded in the Mount Cameroon, Kupe Manengouba and Ndu massifs. These are secondary malaria vectors in forested areas [1, 2, 62, 63] even if *An. funestus* (*s.s.*) has been reported as primary malaria vector in Savannah areas [29, 30, 64]. The presence of *An. melas* in the coastal plain (Tiko) confirms its association with saltwater where its larvae develop [2, 61]. *Anopheles arabiensis* occurred exclusively in the Sudan-Savannah domain (Mbaw plain), in agreement with literature data [52, 61, 65].

Overall, the anopheline fauna were more frequently caught outdoors (73.6%) than indoors (26.4%). However, the biting behaviour should be considered for each *Anopheles* spp. in each wetland as there was considerable variation. For example, in Kumba and Mamfe, both *An. gambiae* and *An. coluzzii* were exclusively exophagic, while in Meanja there was both indoor and outdoor biting of these sibling species. To calculate the extent of transmission that occurs outdoors, it is important to consider the location of the human population during peak biting times, because if biting is primarily nocturnal then the majority of the humans may be indoors. This was demonstrated in south Cameroon where *An. gambiae* (*s.l.*) was found both outdoors and indoors [11], although 52–85% of *An. gambiae* (*s.l.*) bites occurred indoors as observed by Antonio-Nkondjio et al. [26] when studying malaria transmission dynamics. On the other hand, *An. funestus* (*s.l.*) has a preference for indoor-feeding but a significant percentage of bites occur outdoors [66] as well as *An. ziemanni* [5]. Killeen et al. [67] had recently shown that although most outdoor malaria transmission is due to a behavioural resistance mechanism, each individual mosquito does have opportunities to enter houses during the extrinsic incubation period and could be potentially exposed to indoor control tools. However, further studies are urgently needed in Kumba and Mamfe in order to assess the extent of outdoor transmission and potential vector control measures that could be implemented in those areas.

*Anopheles gambiae* and *An. coluzzii* showed a marked nocturnal activity with intense human bites taking place between 22:00 h and 4:00 h while *An. funestus* (*s.s.*) was mostly caught from 1:00 to 6:00 h. These observations are consistent with others studies [1, 11, 66] which reported that members of the *An. gambiae* complex and *An. funestus* group mostly bite late at night, between midnight and the early hours of the morning (00:00–5:00 h), contrasting with *An. ziemanni* whose biting activity declined throughout the night with a peak period early in the evening until midnight (18:00–00:00 h). This later species when in sympatry with *An. gambiae* could ensure an increased risk of malaria transmission as previously observed in Ethiopia by Kenea et al. [54]. However, there is no evidence of that the early biting behaviour observed in *An. ziemanni* in this study or in others is a result of behavioural modifications or shifts.

This study revealed relatively high parity rates of female *Anopheles* (≥ 70%) in the study area, except the Meanja site (≤ 55%) in the Mount Cameroon massif. These variations underline the great variability of parity rates observed sometimes between study sites, and within the same site depending on the species density dynamics. High parous rates could be influenced by temporal fluctuations such as the beginning of rainfall coupled to the emergence of nulliparous individuals (for example, in Tiko, Meanja and Santchou, mosquitoes were collected after the dry period) or associated with the non-application of control measures, thus leading to an accumulation of older populations with time [68], especially in the Ndop plain where *An. gambiae* had the highest life expectancy (28.47 days). The parous ratio and longevity of *An. gambiae* in Santchou was comparable to data obtained by Tchuinkam et al. [4] when studying the bionomics of *Anopheles* species and malaria transmission in the same area. Alternatively, the low parous rate and consequently the decrease in longevity of *An. coluzzii* in Meanja, could be further investigated and associated to the application of control measures and malaria endemicity level in this area.

Overall, the transmission of malaria in the wetlands of the volcanic chain of Cameroon seemed to be low as shown by an overall EIR ranging from 0.23 to 2.24 infected bites per person per month. Although *An. coluzzii* was the most commonly found biting humans and its sibling *An. gambiae* was the most frequently tested positive to *Plasmodium* infection, infection rates showed no significant difference between anophelines tested positive for *Plasmodium* infection. These observations are in-line with that of Gnémé et al. [69] who showed that there was no difference between *An. coluzzii* and *An. gambiae* in their infection susceptibility to *Plasmodium falciparum*. Moreover, *An. gambiae*, generally known to be the main malaria vector in most areas of the southern forested parts of the country [2, 70], played a secondary role in Ndop. The contribution of *An. ziemanni* in the Ndop plain (0.7 infected bites per month) is comparable with values obtained by Tabue et al. [5] in the same area, and comparable with its low anthropophagy as previously shown [71, 72]. However, this species has been previously reported to play a secondary role in the transmission of malaria in several eco-epidemiological settings of Cameroon [2, 9].

## Conclusions

To our knowledge, this study represents the most formal and extensive work available to date of *Anopheles* species in wetlands along the lower section of the volcanic chain of Cameroon. Eight *Anopheles* species were present across the study area, all known to be potential malaria vectors in Cameroon. *Anopheles gambiae* was widespread and probably the main vector in the land, whereas in coastal areas *An. coluzzii* dominated. These dominant vector species were relayed by other species of *Anopheles*, sometimes of considerable local importance such as *An. ziemanni* in the Ndop plain. The unequal distribution of the *Anopheles* species within the studied wetlands, may further confirm that the occurrence of these mosquitoes truly varies according to macro- and microenvironmental differences exhibited by different bio-ecological areas. Most of the host-seeking vectors showed a preference for feeding outdoors rather than indoors which could be a challenge to the malaria control strategies which uses mainly predominantly indoor control intervention tools such as long-lasting insecticidal nets (LLINs) and indoor residual spraying (IRS). Of further challenge is the observation that these mosquitoes were found biting early in the evening as well as late in the night depending on the species when people might not be protected by LLINs. A regular update of vector species is thus necessary for effective malaria control.

## Additional files

Additional file 1: **Figure S1.** Cameroon volcanic line. (PDF 405 kb)

Additional file 2: **Table S1.** Summary information of the surveyed wetlands and environmental factors. (PDF 194 kb)

Additional file 3: **Figure S2.** Flow chart of activities. (PDF 47 kb)

Additional file 4: **Table S2.** Sampling parameters for mosquito collections. (PDF 185 kb)

Additional file 5: **Table S3.** Two-way ANOVA statistical significance for each outdoor-biting *Anopheles* species within and between wetlands. (PDF 212 kb)

## Abbreviations

EIR: Entomological inoculation rate
IR: Infection rate
MBR: Man-biting rate
P: Probability of daily survival
PR: Parity rate or parous ratio
