# Supplementary figures and images for: Bionomics and vectorial role of anophelines in wetlands along the volcanic chain of Cameroon
Source: Parasit Vectors. 2018 Aug 14;11:471. doi: 10.1186/s13071-018-3041-z (PMC6092805; doi:10.1186/s13071-018-3041-z)

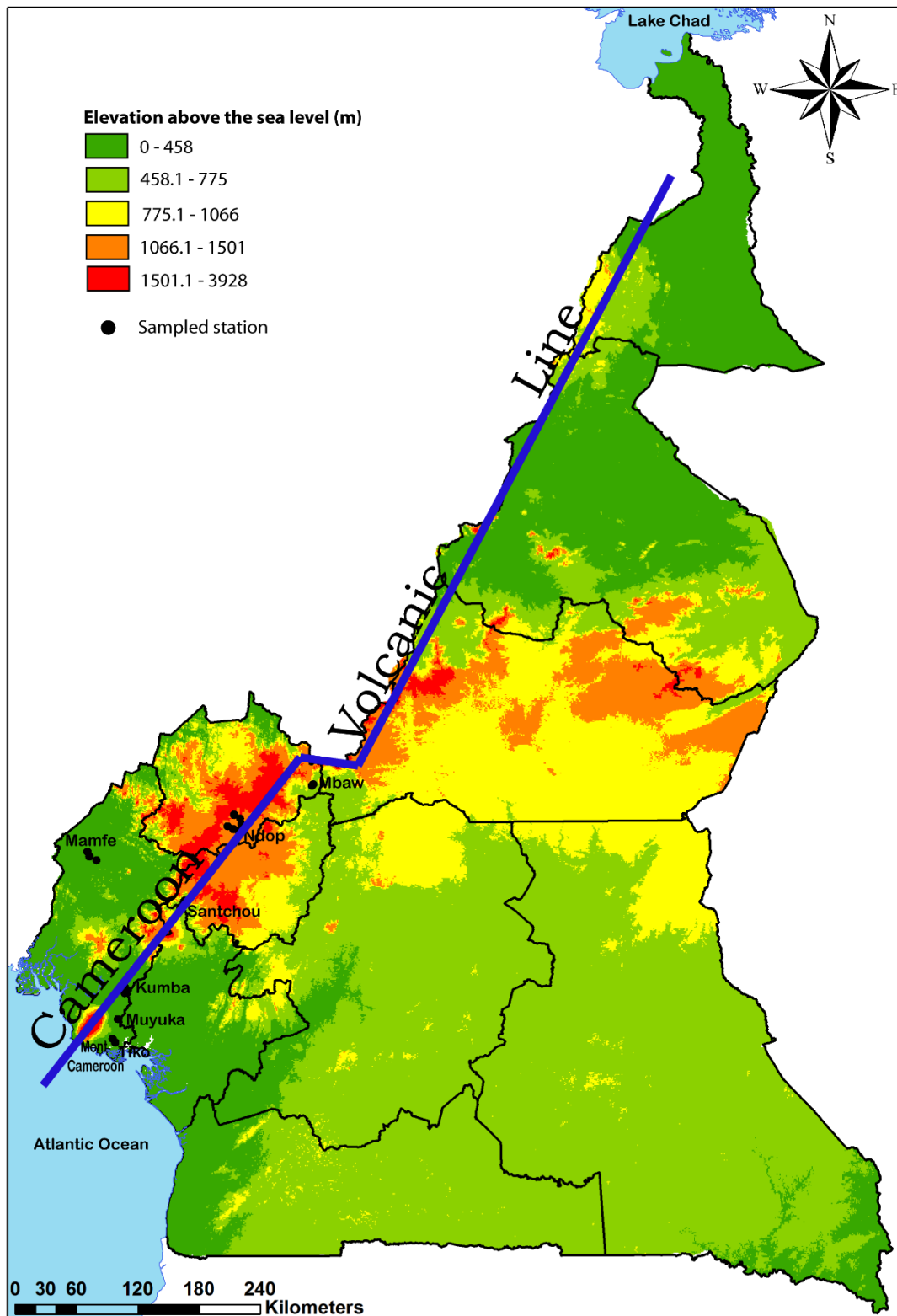

Additional file 1: Figure S1. Cameroon volcanic line.

Supplement: Supplementary file 1 — Figure S1. Cameroon volcanic line. (PDF 405 kb) [file 13071_2018_3041_MOESM1_ESM.pdf]

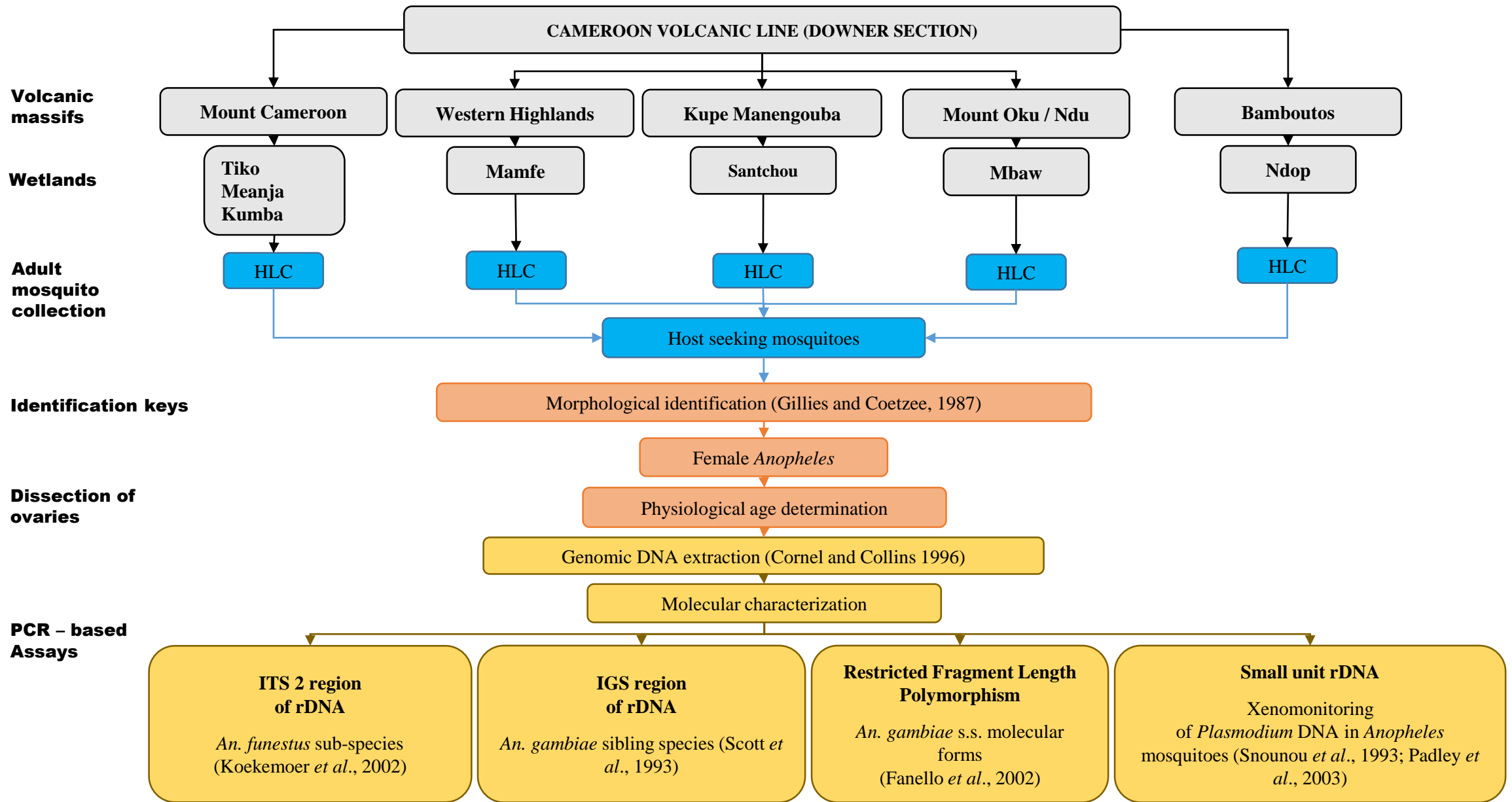

Additional file 3: Figure S2. Flow chart of activities

Supplement: Supplementary file 3 — Figure S2. Flow chart of activities. (PDF 47 kb) [file 13071_2018_3041_MOESM3_ESM.pdf]
