# Supplementary material for: Bionomics and vectorial role of anophelines in wetlands along the volcanic chain of Cameroon
Source: Parasit Vectors. 2018 Aug 14;11:471. doi: 10.1186/s13071-018-3041-z (PMC6092805; doi:10.1186/s13071-018-3041-z)
Supplement: Supplementary file 2 — Table S1. Summary information of the surveyed wetlands and environmental factors. (PDF 194 kb) [file 13071_2018_3041_MOESM2_ESM.pdf]

Additional file 2: Table S1. Summary information of the surveyed wetlands and environmental factors

wetland	Topography	Number of inhabitants	Average minimum and maximum temperature	Annual precipitation	Data on *P. falciparum* prevalence rate	Economic activities
Tiko	Coastal lowland with many marshy areas and mangrove swamps	134,649	25°C – 32°C	2975 mm	39%[a]	Agro-pastoral, forest exploitation, industrial farms (rubber, banana, palm oil)
Kumba	Inland urban area (wetland of international importance) with the Barombi Mbo, a large crater lake being the most geographical attraction	265,071	22.2°C – 29.3°C	2751 mm	72.3%[b]	Agriculture (cocoa, rubber and banana production) trading
Meanja	Gentle slopes and lowlands with bog, marsh and swamp vegetation	7,296	23°C – 33°C	3037 mm	62%[c]	Agriculture
Mamfe	High relief consisting of mountain ranges and volcanoes with the presence of many volcanic lakes	34,225	22°C – 31.6°C	2761 mm	Not available	Agro-pastoral and forest exploitation
Santchou	Juxtaposition of small hills drawn together by little streams flowing down towards swampy lakes carrying Shrubby savannah with isolated woodland	37,479	15.5°C – 26°C	1950 mm	29.3%[d]	Agriculture
Ndop	Inland fresh water wetland surrounded by hills and remarked by riverine floodplains, marshes and an artificial lake	200,000	14°C – 28°C	1904 mm	19.3%[e]	Agriculture (swamp rice cultivation), grazing, hunting and fishing
Mbaw plain	Ferruginous soils being essentially derivatives of granitic deposits. There are equally volcanic soils of the basaltic type with depositional formations of alluviums and unsorted swamp.	121,642	17.1°C – 28.6°C	2150 mm	Not available	Agriculture (maize, beans, yam, vegetable, coffee, rice)

[a] Bigoga J, Manga L, Titanji V, Coetzee M, Leke R. Malaria vectors and transmission dynamics in coastal south-western Cameroon. Malar J. 2007; 6 5; doi: 10.1186/1475-2875-6-5.
[b] Njunda AL, Ngouadjeu DTE, Nsagha DS, Nyanjoh EM, Kwenti TD, Assob NJC. Haematological profile of children with malaria in Kumba Health District, South West Region Cameroon. IJIH. 2016; 6: 23-9.
[c] Eyong EEJ, Kengne-Ouafo AJ , Chounna NWP , Datchoua-Poutcheu FR, Wanji S. Altitudinal variation in the parasitological and entomological indices of malaria around Mount Cameroon, South West Region of Cameroon. JPVB. 2016; 8 8: 74-85; doi: 10.5897/JPVB2016.0242.
[d] Mangham LJ, Cundill B, Achonduh OA, Ambebila JN, Lele AK, Metoh TN, Ndive SN, Ndong IC, Nguela RL, Nji AM, Orang-Ojong B, Wiseman V, Pamen-Ngako, J, Mbacham WF. Malaria prevalence and treatment of febrile patients at health facilities and medicine retailers in Cameroon. Tropical Medicine & International Health. 2012; 17: 330-42.
[e] Sumo L, Mbah EN, Nana-Djeunga HC. Malaria in pregnancy in the Ndop health district (North West Region, Cameroon): results from retrospective and prospective surveys. JPVB. 2015; 7 9: 177-81; doi: 10.5897/JPVB2015.0214.
