# Supplementary material for: Bionomics and vectorial role of anophelines in wetlands along the volcanic chain of Cameroon
Source: Parasit Vectors. 2018 Aug 14;11:471. doi: 10.1186/s13071-018-3041-z (PMC6092805; doi:10.1186/s13071-018-3041-z)
Supplement: Supplementary file 4 — Table S2. Sampling parameters for mosquito collections. (PDF 185 kb) [file 13071_2018_3041_MOESM4_ESM.pdf]

Additional file 4: Table S2. Sampling parameters for mosquito collections

Volcanic massifs	Wetlands	Number of days of collection	Number of sample stations	Number of man-nights
Mount Cameroon	Tiko	5	2	40
	Kumba	5	2	40
	Meanja	5	1	20
Western highlands	Mamfe	5	3	60
Kupe Manengouba	Santchou	5	5	100
Oku/Ndu	Mbaw	5	2	40
Bamboutos	Ndop	5	6	120
Total		35	21	420
