# Supplementary material for: Bionomics and vectorial role of anophelines in wetlands along the volcanic chain of Cameroon
Source: Parasit Vectors. 2018 Aug 14;11:471. doi: 10.1186/s13071-018-3041-z (PMC6092805; doi:10.1186/s13071-018-3041-z)
Supplement: Supplementary file 5 — Table S3. Two-way ANOVA statistical significance for each outdoor-biting Anopheles species within and between wetlands. (PDF 212 kb) [file 13071_2018_3041_MOESM5_ESM.pdf]

Additional file 5: Table S3. Two-way ANOVA statistical significance for each outdoor-biting *Anopheles* species within and between wetlands.

Wetlands		Tiko			Meanja		Kumba		Mamfe		Santchou		Ndop			Mbaw	
	***Anopheles* species**	*An. coluzzii*	*An. melas*	*An. funestus* s. s.	*An. coluzzii*	*An. funestus* s. s.	*An. coluzzii*	*An. gambiae*	*An. coluzzii*	*An. gambiae*	*An. gambiae*	*An. nili*	*An. gambiae*	*An. ziemanni*	*An. hancocki*	*An. gambiae*	*An. arabiensis*
**Tiko**	*An. coluzzii*	-															
	An. melas	*	-														
	An. funestus s. s.	**	ns	-													
**Meanja**	*An. coluzzii*	ns	ns	ns	-												
	An. funestus s. s.	**	ns	ns	ns	-											
**Kumba**	*An. coluzzii*	**	ns	ns	ns	ns	-										
	An. gambiae	ns	**	***	**	***	***	-									
**Mamfe**	*An. coluzzii*	**	ns	ns	ns	ns	ns	***	-								
	An. gambiae	ns	ns	**	ns	*	*	ns	*	-							
**Santchou**	*An. gambiae*	*	ns	ns	ns	ns	ns	***	ns	ns	-						
	An. nili	**	ns	ns	ns	ns	ns	***	ns	*	ns	-					
**Ndop**	*An. gambiae*	**	ns	ns	ns	ns	ns	***	ns	*	ns	ns	-				
	An. ziemanni	ns	ns	ns	ns	ns	ns	*	ns	ns	ns	ns	ns	-			
	An. hancocki	**	ns	ns	ns	ns	ns	***	ns	*	ns	ns	ns	ns	-		
**Mbaw**	*An. gambiae*	ns	ns	*	ns	*	*	ns	*	ns	ns	*	*	ns	*	-	
	An. arabiensis	**	ns	ns	ns	ns	ns	***	ns	*	ns	ns	ns	ns	ns	*	-

*p < 0.05, **p < 0.001, ***p < 0.0001, ns: not significant.
